# A Retrospective Cohort Study of the Impact of Nurse Practitioners on Hospitalized Patient Outcomes

**DOI:** 10.3390/nursrep11010003

**Published:** 2021-01-06

**Authors:** Manish S. Patel, Lauren C. Hogshire, Helaine Noveck, Michael B. Steinberg, Donald R. Hoover, Jane Rosenfeld, Akanksha Arya, Jeffrey L. Carson

**Affiliations:** 1 Division of General Internal Medicine, Department of Medicine, Rutgers Robert Wood Johnson Medical School, New Brunswick, NJ 08901, USA; lauren.hogshire@pennmedicine.upenn.edu (L.C.H.); noveck@rwjms.rutgers.edu (H.N.); steinbmb@rwjms.rutgers.edu (M.B.S.); rosenfja@rwjms.rutgers.edu (J.R.); carson@rwjms.rutgers.edu (J.L.C.); 2 Department of Statistics and Institute for Health, Healthcare Policy and Aging Research, Rutgers University, Piscataway, NJ 08854, USA; drhoover@stat.rutgers.edu; 3 Department of Medicine, Sidney Kimmel Medical College, Thomas Jefferson University, Philadelphia, PA 19107, USA; axa395@jefferson.edu

**Keywords:** nurse practitioner, inpatient, outcomes, quality of care, hospital

## Abstract

The role of advanced practice providers has expanded in the hospital setting. However, little data exist examining the impact of these providers. Our purpose was to determine the effect of adding nurse practitioners in a complementary role on the quality and efficiency of care of hospitalized patients. A retrospective cohort study evaluated adult patients admitted by private physicians (without house staff or non-physician providers) to a general medical-surgical unit in an academic medical center. The admissions department allocated patients as beds became available and nurse practitioners were assigned to patients until their caseload was reached. Outcomes included length of hospital stay, in-hospital mortality, admission costs, 30-day readmissions, transfer to a more intensive care level, and discharge order time. Of the 382 patients included in this study, 263 were assigned to the nurse practitioner group. Hospital mortality was lower in the nurse practitioner group [OR 0.11 (95% CI 0.02–0.51)] as was transfer to more intensive care level [OR 0.39 (95% CI 0.20–0.75)]; however, the nurse practitioner group had longer length of stay (geometric mean = 5.80 days for nurse practitioners, 3.63 days for no nurse practitioners; *p* < 0.0001) and higher cost per patient (geometric mean = USD 6631 vs. USD 5121; *p* = 0.005). The results were unchanged when models were adjusted for potential confounders. Adding nurse practitioners can yield improved clinical outcomes (lower hospital mortality and fewer transfers to intensive care), but with a potential economic expense (longer hospital stays and higher costs).

## 1. Introduction

As healthcare costs have skyrocketed, system changes are critically needed to improve care delivery while keeping costs in check. One of the most common methods to accomplish this goal has been the utilization of advanced practice providers, such as nurse practitioners.

The role of nurse practitioners and other advanced practice providers has expanded in all healthcare settings, but especially in hospitals. Contemporary data from the American Academy of Nurse Practitioners revealed that there were over 290,000 licensed nurse practitioners in the U.S. [1] About one-third of all nurse practitioners practice in a hospital setting [2]. The proliferation of hospital-based nurse practitioners can be attributed to resident work-hour restrictions, shortages of certain providers, such as intensivists, desire for improved continuity and access to care, demand for high-quality cost-effective care, and the growth of hospitalist programs nationwide [3].

The roles of nurse-practitioners and other advanced practice providers can be classified into two broad categories: alternative and complementary [4,5]. In an alternative role, nurse practitioners perform similar or identical services as physicians, in an effort to increase the volume of patients seen, decrease physician workload, and lessen costs. Nurse practitioners used in a complementary role provide additional services that supplement services provided by physicians, in order to broaden types of services given to patients and enhance the quality of care.

Limited evidence regarding the effectiveness of nurse practitioners in the hospital setting demonstrates conflicting results and varied methodology. A systematic review revealed only one randomized controlled trial of nurse practitioners in the adult medical-surgical setting [5]. This trial compared resident physicians with nurse practitioners in an alternative role and found no differences in cost, length of stay, or mortality between the two groups [6]. In another quasi-experimental trial, nurse practitioners were added in a complementary role to resident medical teaching teams, which resulted in a lower length of stay and reduced costs, but no difference in mortality or readmissions [7]. Several other observational studies conducted on inpatients utilized nurse practitioners in an alternative role to resident physician care and showed longer lengths of stay and higher costs in the nurse practitioner groups, but no differences in mortality and readmissions [8,9,10]. There is a dearth of evidence studying nurse practitioners in the hospital setting utilized in a complementary role in adult general medical-surgical patients.

We conducted a retrospective cohort study of nurse practitioners used in a complementary role in an inpatient, adult, medical-surgical population and evaluated the impact of nurse practitioners on patient outcomes, patient flow, and costs compared with a control group of patients cared for by their private physicians alone.

## 2. Methods

### 2.1. Design and Participants

We performed a retrospective cohort study that evaluated adult patients admitted to a general medical-surgical unit of a single university hospital from 1 March 2014 to 31 October 2014. We included adult patients assigned by the admissions department based on bed availability and as per the hospital’s usual process to a medical-surgical bed on the unit at the time of hospital admission. We only included patients on private physician services who were not covered by resident or advanced practice providers. We excluded patients from the study if they were transferred to the unit from elsewhere in the hospital, if they were on teaching services or private physician/hospitalist services that employed advanced practice providers, or if the patients were admitted directly to the intermediate care (step-down) beds on the unit. Subsequent hospital admissions for the same individual were excluded, although they could be counted as outcomes. Finally, patients were excluded if they remained in the hospital past 31 October 2014. All the study data were taken from hospital administrative databases. The Rutgers University Institutional Review Board deemed this study exempt.

### 2.2. Intervention

Two nurse practitioners were assigned to cover consecutively numbered beds until their caseload of 14 patients each was reached. Each time the caseload dropped to under 14 patients, the nurse practitioner would add additional patients to maintain their full caseload. We compared the intervention group of private patients cared for by the nurse practitioners to a control group of private patients cared for by their attending physicians without the assistance of the nurse practitioners. The nurse practitioners were masters prepared with over five years of experience as nurse practitioners in both inpatient and outpatient settings. Prior to this they both had over 22 years of experience as registered nurses, more than 20 of which were in the intensive care unit (ICU) setting.

The nurse practitioners caring for patients in the intervention group functioned in a complementary role to the attending physicians. These nurse practitioners did not write progress notes, perform admitting history and physical examinations, or write discharge summaries. The primary attending physicians would perform rounds on the patients, write history and physical examinations and daily progress notes, and place orders as per their usual customs. The nurse practitioners would handle urgent issues and routine problems, delivering services in person, such as examining patients, when the primary attending physician was not available to do. They were available to place orders, including discharge orders, if needed. They would also participate in multidisciplinary rounds with floor nurses, case managers, and social workers to coordinate discharge plans. The attending physicians generally did not attend these multidisciplinary rounds. The nurse practitioners communicated with the primary attending physician and consultants as needed.

### 2.3. Outcomes

We examined the following outcomes: in-hospital mortality, length of hospital stay, direct cost of the admission (excluding room and board), readmissions within 30 days, and transfer to more intensive level of care (intermediate/step-down or ICU). We also evaluated whether the discharge order was written by 11:00 a.m., which was an administrative goal of the hospital.

### 2.4. Data Analysis

We compared baseline patient characteristics and study outcomes between study subjects in each study arm using odds ratios, relative risks, absolute differences with Chi-squared tests of statistical significance for categorical variables, and arithmetic differences with *t*-tests for continuous variables, and nonparametric tests of significance for continuous variables with markedly non-normal distributions. Study outcomes between the two arms were similarly compared. Comparisons of outcomes were further adjusted for potential confounding using multivariate models (logistic regression for categorical and linear regression for continuous), that included age, gender, admitting diagnosis, and the Charlson Co-Morbidity Index calculated utilizing ICD-9 codes. Due to the skewness of the data, length of stay and cost of the admission were log-transformed and expressed as geometric means.

## 3. Results

### 3.1. Participant Characteristics

From a total of 760 private physician admissions, we included 382 patients in the study; 263 patients were covered by nurse practitioners in the intervention group, and 119 patients served as the control group. We excluded 378 patients because they were transferred into this medical-surgical unit from elsewhere in the hospital. Baseline characteristics were similar between the nurse practitioner and control groups (Table 1).

Overall, 55.5% of the participants were male, with more than 70% being over 65 years old. In addition, there were a wide range of co-morbidities; the most common underlying disorders were cardiac (23%), pulmonary (18%), and gastroenterological (13%) diagnoses. About one-third of patients had a Charlson Co-Morbidity Index of 0.

### 3.2. Patient Outcomes

In-hospital mortality was lower in the nurse practitioner intervention group. There were two deaths (0.8%) in the intervention group versus eight deaths (6.7%) in the control group [Odds Ratio (OR) 0.11 (95% CI 0.02–0.51); Relative Risk (RR) 0.11 (95% CI 0.02–0.53); Arithmetic Difference (AD) −6.0% (95% CI −10.5% to −1.3%)] (Table 2).

Patients in the nurse practitioner intervention group were also less likely to be transferred to a more intensive level of care. 7.2% of patients in the intervention group were transferred to more intensive care during the admission versus 16.8% of patients in the control group [OR 0.39 (95% CI 0.20–0.75); RR 0.43 (95% CI 0.29–0.78); AD −9.6% (95% CI −17.0% to −2.2%)].

The length of the hospital stay, however, was longer in the intervention group. Those patients in the nurse practitioner group had a geometric mean length of stay of 5.80 days (95% CI 5.29–6.36 days), compared with a geometric mean of 3.63 days (95% CI 3.13–4.22 days) in the control group [Ratio of Geometric Mean 1.60 (95% CI 1.35–1.89), *p* < 0.0001] (Table 3).

Patients in the nurse practitioner group also had higher direct costs of admission per patient. For those in the intervention group, the geometric mean cost of admission per patient was USD 6631 (95% CI USD 6023–7300), compared with a geometric mean of USD 5121 (95% CI USD 4372–5999) in the control group [Ratio of Geometric Mean 1.29 (95% CI 1.08–1.55), *p* = 0.005] (Table 3).

There were no statistical differences in the number of readmissions within 30 days between the two groups; 15.2% of the patients in the intervention group were readmitted within 30 days of discharge, versus 13.4% of patients in the control group [OR 1.15 (95% CI 0.62–2.16); RR 1.13 (95% CI 0.66–1.94); AD −2.6% (95% CI −11.9% to 6.7%)]. Similarly, there were no statistical differences in discharge order times between the two groups. The discharge order outcome was limited to those patients discharged alive from the hospital and where a discharge order was present, resulting in a total number of 365 patients. A discharge order was written by 11:00 AM in 20.3% of patients in the nurse practitioner intervention group versus 22.9% of patients in the control group [OR 0.86 (95% CI 0.50–1.47); RR 0.89 (95% CI 0.58–1.35); AD 1.8% (95% CI −5.7% to 9.3%)] (Table 2).

Logistic regression models were run to adjust for potential confounding factors, such as age, gender, admitting diagnosis, and Charlson Co-Morbidity Index found qualitatively similar findings for each of these outcomes (Table 2 and Table 3).

## 4. Discussion

We evaluated the clinical and financial impact of two nurse practitioners functioning in a complementary role in hospitalized adult medical-surgical patients and found lower mortality and frequency of transfer to intensive care setting in patients cared for by a nurse practitioner in addition to their attending physician. This clinical benefit was accompanied by longer length of hospital stay and higher cost per admission. There were no differences in 30-day readmissions or discharge order time between the intervention and control groups.

Specifically, patients in the nurse practitioner intervention group were 90% less likely to experience in-hospital mortality, although the overall event rates were low. Similarly, patients covered by the nurse practitioners were also about 60% less likely to be transferred to a more intensive level of care. However, this was associated with longer lengths of stay and higher costs. Those in the nurse practitioner intervention group had hospital stays that were, on average, two days longer than the control group, even when adjusted for co-morbidities, as well as higher costs per hospital stay.

It appears that the higher costs were driven mainly by the longer lengths of stay. Based on the administrative dataset, however, we are unable to discern whether differences in practice patterns of the nurse practitioners or physicians, such as resource utilization, may have contributed to the cost differences, in addition to the length of stay. The reasons for the increased length of stay are similarly unknown.

As opposed to studies conducted in outpatients, a systematic review found only one randomized controlled trial evaluating the role of nurse practitioners in an inpatient, adult, medical-surgical population [5]. Pioro et al. utilized nurse practitioners in an alternative role and randomized 381 patients to nurse practitioner-based care vs. resident-based care [6]. There were no statistical differences between patients cared for by the nurse practitioners compared with residents in the intention to treat analysis with respect to mean length of stay (5.0 vs. 5.3 days, *p* > 0.1), mean total hospital charges (USD 8854 vs. USD 9426, *p* > 0.1), transfer to ICU (3.6% vs. 6.9%, *p* > 0.1), or hospital mortality (1.6% vs. 1.1%, *p* > 0.1). Regarding costs, there were no statistical differences in the mean total ancillary charges between the two groups, including pharmacy, radiology, laboratory, and respiratory therapy costs. There was, however, a high rate of crossover from the nurse-practitioner group to the resident-based care group, which may have biased the results. 

A similarly designed quasi-experimental comparative trial conducted by Cowan et al. added nurse practitioners in a complementary role to teams in adult general medical patients [7]. This study showed that patients covered by nurse practitioners stayed, on average, one day less that the control group (three vs. four days, *p* < 0.001), resulting in an average USD 1707 profit per day per patient realized from the reduction in length of stay. There were no differences in mortality or readmission rates between the two groups. In contrast, the results from our trial showed lower mortality with higher costs and lengths of stay in the nurse practitioner group. This trial differed from ours in that the nurse practitioners were added to resident medical teaching teams, which may affect the results’ applicability to non-academic medical centers and non-resident covered patients.

Observational studies done on adult inpatients utilizing nurse practitioners have yielded mixed results. One retrospective study utilizing nurse practitioners in a complementary role on a surgical service showed the addition of nurse practitioners increased the use of home services and decreased unnecessary emergency room visits [8]. Unlike our study, this study was conducted in a purely surgical population and did not evaluate mortality, readmission rates, length of stay, or costs of admission. Another retrospective trial comparing nurse practitioners used in an alternative role versus resident-led teams in general medical patients found no differences in in-hospital mortality between the two groups, but did reveal that the patients on the nurse practitioner teams had longer lengths of stay and higher per-patient direct costs [9]. A final observational trial utilized a descriptive comparative design and compared nurse practitioner-led care in an alternative role versus resident physician-led care in 100 geriatric inpatients [10]. As compared to nurse practitioners, those patients cared for by the physicians had lower lengths of stays and costs, with no differences in mortality and readmission rates. It should be noted, however, that the patients cared for by the nurse practitioners tended to be older and of a higher acuity than those cared for by the residents [10]. Although the length of stay and cost results for the two latter studies are in line with our results, the use of nurse practitioners in alternative roles, the comparison to resident physician care, and the differences in baseline patient characteristics limit the direct comparability to our study.

Other systematic reviews have included largely observational trials [4,11,12]. One systematic review evaluated trials and prior reviews of nurse practitioners used in a complementary role across various practice settings and patient types [4]. The authors concluded that nurse-led care was associated with a decreased readmission rate, but more tests were ordered. No conclusions could be drawn on mortality or overall healthcare costs. Additional systematic reviews did not differentiate between alternative and complementary roles for the nurse practitioners, suffered from low quality of evidence, had conflicting conclusions regarding length of stay, and showed no differences in mortality or costs with the addition of nurse practitioners [11,12].

Our study had several limitations. Firstly, this was a single hospital study with a modest sample size and a small number of outcomes. These study characteristics increase the probability that the results may not be replicated in a larger clinical trial. This could also explain the large mortality effect of the intervention. We were also limited to the hospital administrative dataset, which meant that we could not extract more detailed data regarding utilization of specific tests and other resources by the nurse practitioners or physicians. Although the medical and administrative data were collected as per the hospital’s usual procedures, inaccuracies in the collection of those data are possible. Similarly, although patients were assigned to beds as they became available using the hospital’s usual process, this was not randomly done and may have introduced selection bias. Finally, this was a retrospective observational cohort study, and there may have been unmeasured confounding factors. The patients, however, were well matched between the two groups, which limited selection bias.

The strengths of our study are that the study population of general medical-surgical patients cared for by individual, private physicians makes the results of this study generalizable to a variety of inpatient settings, including non-academic medical centers. Our study adds valuable outcomes data to the very sparse available evidence regarding the complementary use of inpatient nurse practitioners. Furthermore, ours is one of the only studies showing a mortality benefit in an adult inpatient general medical-surgical population.

## 5. Conclusions

In conclusion, the addition of nurse practitioners in a complementary role to adult general medical-surgical inpatients improved clinical outcomes with lower in-hospital mortality and fewer transfers to a more intensive level of care. These benefits were offset with longer lengths of stay and higher costs. These findings require confirmation in large, randomized trials, conducted in varied settings, to evaluate the outcomes and cost-effectiveness of utilizing nurse practitioners for hospitalized patients 

## Figures and Tables

**Table 1:** Baseline Characteristics.

	Nurse Practitioner *n* = 263		Control Group *n* = 119		Overall *n* = 382		*p*-Value
	*n* (%)		*n* (%)		*n* (%)		
**Gender**							0.38
Male	150	(57.0)	62	(52.1)	212	(55.5)	
Female	113	(43.0)	57	(47.9)	170	(44.5)	
**Age**							0.51
Less than 65	72	(27.4)	41	(34.5)	113	(29.6)	
65 to 75	66	(25.1)	30	(25.2)	96	(25.1)	
76 to 84	60	(22.8)	22	(18.5)	82	(21.5)	
85 and older	65	(24.7)	26	(21.8)	91	(23.8)	
**Charlson Co-Morbidity Index**							0.28
0 points	88	(33.5)	36	(30.3)	124	(32.5)	
1 point	41	(15.6)	30	(25.2)	71	(18.6)	
2 points	54	(20.5)	21	(17.6)	75	(19.6)	
3 points	31	(11.8)	14	(11.8)	45	(11.8)	
4 or more points	49	(18.6)	18	(15.1)	67	(17.5)	
**Primary Admitting Diagnosis**							
Infectious Disease	12	(4.6)	6	(5.0)	18	(4.7)	0.80
Neoplasm	13	(4.9)	7	(5.9)	20	(5.2)	0.80
Endocrine Disorder	14	(5.3)	5	(4.2)	19	(5.0)	0.80
Hematologic Disorder	10	(3.8)	2	(1.7)	12	(3.1)	0.35
Mental Disorder	1	(0.4)	2	(1.7)	3	(0.8)	0.23
Neurologic Disease	7	(2.7)	3	(2.5)	10	(2.6)	1.00
Cardiovascular Disease	62	(23.6)	26	(21.8)	88	(23.0)	0.79
Respiratory Disease	45	(17.1)	25	(21.0)	70	(18.3)	0.39
Gastrointestinal Disease	37	(14.1)	13	(10.9)	50	(13.1)	0.51
Genitourinary Disease	22	(8.4)	9	(7.6)	31	(8.1)	1.00
Skin and Subcutaneous Disease	12	(4.6)	5	(4.2)	17	(4.5)	1.00
Musculoskeletal and Connective Tissue Disease	7	(2.7)	6	(5.0)	13	(3.4)	0.24
Injury and Poisoning	21	(8.0)	10	(8.4)	31	(8.1)	1.00

**Table 2:** Outcome Measures of Dichotomous Variables.

Outcome	Nurse Practitioner *n* (%)	Control *n* (%)	Unadjusted OR (95% CI)	Adjusted OR (95% CI) *
In-hospital mortality	2 (0.8)	8 (6.7)	0.11 (0.02–0.51)	0.09 (0.02–0.44)
Transfer to more intensive care level	19 (7.2)	20 (16.8)	0.39 (0.20–0.75)	N/A
30-day readmission	40 (15.2)	16 (13.4)	1.15 (0.62–2.16)	1.13 (0.60–2.12)
Discharge order written by 11AM [limited to discharged alive (*n* = 372) and 7 missing orders (Final *n* = 365)]	52 (20.3)	25 (22.9)	0.86 (0.50–1.47)	N/A

OR, odds ratio; CI, confidence interval. * Stepwise modeling for potential confounders (age, gender, Charlson Co-Morbidity Index, admitting diagnosis): In-hospital death-retained Charlson Co-Morbidity Index in final model. Transfer to more intensive care—no potential confounders in final model. Readmission within 30 days resulted in a retained Charlson Co-Morbidity Index. Discharge order written by 11 a.m.—no potential confounders in final model.

**Table 3:** Outcomes Measures of Continuous Variables.

Outcome	Nurse Practitioner GM (95% CI)	Control GM (95% CI)	Ratio of GM (NP/Control)	
			Unadjusted (95% CI)	Adjusted (95% CI) *
Length of Stay (days)	5.80 (5.29–6.36)	3.63 (3.13–4.22)	1.60 (1.35–1.89)	1.58 (1.34–1.86)
Cost of Admission (USD)	6631 (6023–7300)	5121 (4372–5999)	1.29 (1.08–1.55)	1.28 (1.07–1.52)

GM, geometric mean; CI, confidence interval; NP, nurse practitioner; USD, United States Dollar. * Stepwise modeling for potential confounders (age, gender, Charlson Co-Morbidity Index, admitting diagnosis): Both outcomes retained Charlson Co-Morbidity Index in final model.

## Data Availability

Data were obtained from Robert Wood Johnson University Hospital and are available from the authors with the permission of Robert Wood Johnson University Hospital.
